# Scarring Patterns in Cutaneous Sporotrichosis: Clinical and Therapeutic Correlations

**DOI:** 10.7759/cureus.114892

**Published:** 2026-08-20

**Authors:** Karina Calbucci, John V Veasey, Elisete I Crocco

**Affiliations:** 1 Dermatology, Irmandade da Santa Casa de Misericórdia de São Paulo, São Paulo, BRA; 2 Dermatology, Santa Casa de São Paulo School of Medical Sciences, São Paulo, BRA; 3 Dermatology, Santa Casa de São Paulo Hospital, São Paulo, BRA

**Keywords:** cryotherapy, dermatology, hypertrophic scar, scar, sporotrichosis

## Abstract

Background: Zoonotic sporotrichosis caused by Sporothrix brasiliensis has emerged as a major public health problem in Brazil, with a growing burden of cutaneous sequelae. However, scarring outcomes remain poorly characterized, particularly regarding specific morphological patterns and clinical factors that influence tissue repair.

Methods: This retrospective descriptive-analytical study included patients with cutaneous sporotrichosis treated at a tertiary referral dermatology center between January 2017 and June 2024. Scars were evaluated individually according to trophism (atrophic, normotrophic, hypertrophic) and specific morphology (linear, "wax drop", or nonspecific), as well as anatomical site, clinical form, presence of inoculation chancre, orientation in relation to skin tension lines and use of cryotherapy. Statistical analyses were performed using Microsoft Excel (Microsoft Corporation, Redmond, WA, USA), with confirmatory re-analysis in Python 3 (SciPy 1.x).

Results: A total of 45 patients (29 (64.4%) female; mean age 35.9 ± 23.8 years) and 202 scars were analyzed. Most scars were located on the upper limbs (162, 80.2%). Overall, 104 (51.5%) were atrophic, 91 (45.0%) hypertrophic, and 7 (3.5%) normotrophic; 43 (21.3%) showed a linear pattern and 37 (18.3%) a ‘wax drop’ pattern. At the scar level, trophism was associated with the presence of an inoculation chancre (p = 0.00026) and with a lesion axis parallel to skin tension lines (p < 0.001); the association with clinical form (p = 0.0018) was driven by the seven normotrophic scars and did not extend to hypertrophic scarring (p = 0.67). Thirty-eight scars (18.8%), arising from six patients, were treated with cryotherapy: atrophic scars were proportionally more frequent at the scar level (28 (73.7%) vs. 76 (46.3%); p = 0.0076), but this was not confirmed with the patient as the unit of analysis (59.4% vs. 45.2%; p = 0.45), nor was treatment duration shorter (30.8 vs. 18.8 weeks; p = 0.27). Because scars were unevenly clustered within patients, scar-level p-values are descriptive.

Conclusions: Cutaneous sporotrichosis is associated with a substantial scar burden, frequently with distinctive linear and ‘wax drop’ patterns; the linear arrangement on the upper limbs may be mistaken for self-inflicted injury and is the most directly actionable observation of this series. The presence of an inoculation chancre and a lesion axis parallel to skin tension lines were associated with hypertrophic scarring at the scar level, whereas differences associated with cryotherapy were not confirmed at the patient level and should not be interpreted as evidence of a therapeutic effect. Prospective studies with clustering-adjusted analyses are needed to validate these observations.

## Introduction

Sporotrichosis is a subcutaneous mycosis whose epidemiological profile has changed markedly in recent decades, particularly in Brazil, where zoonotic transmission associated with Sporothrix brasiliensis has led to sustained urban outbreaks [1-4]. Beyond the infectious process itself, the disease frequently leaves permanent cutaneous sequelae, which may cause functional, aesthetic, and psychosocial impairment [5].

While clinical forms, diagnosis and antifungal treatment of sporotrichosis have been extensively described [6-10], post-treatment scarring has received comparatively little attention. Linear scars and other distinctive scar appearances following sporotrichosis, including their potential diagnostic value, have already been described [11]. However, these observations have not yet been expanded into a structured analysis of scar trophism and morphology, nor systematically correlated with clinical and therapeutic determinants.

Despite increasing recognition of zoonotic sporotrichosis as an emerging public health problem in Brazil, the long-term cutaneous sequelae of the infection remain insufficiently characterized. Understanding the patterns and determinants of scarring may provide clinically relevant information for patient counseling and for strategies aimed at reducing permanent morbidity.

Taken together, current knowledge is largely limited to the observation that permanent scars occur after cutaneous sporotrichosis and that linear patterns may aid diagnosis [11]. What remains uncharacterized is the distribution of scar trophism, the frequency of the “wax drop” morphology, and the degree to which these outcomes correlate with clinical form, presence of an inoculation chancre, lesion orientation relative to skin tension lines, and adjunctive cryotherapy.

The present study aimed both to characterize scarring patterns following cutaneous sporotrichosis and to assess their association with clinical presentation, lesion topography, and therapeutic approach.

## Materials and methods

Study design and population

A retrospective descriptive-analytical study was conducted using medical records from patients with confirmed sporotrichosis treated at the Tropical Dermatoses Outpatient Clinic of the Dermatology Service of Santa Casa de São Paulo between January 2017 and June 2024. The diagnosis was established by a compatible clinical and epidemiological presentation associated with laboratory confirmation, and all included patients had isolation of Sporothrix spp. in culture and/or histopathological findings consistent with the disease; no patient was included on clinical-epidemiological grounds alone. Patients of any age or sex who completed treatment and had complete clinical records and photographic documentation at diagnosis and at clinical cure were eligible. All consecutive eligible patients treated during the study period were included, without further sampling; the only exclusion criteria were incomplete clinical records or absence of standardized photographic documentation at diagnosis and at clinical cure.

Clinical form

The clinical form was recorded for each lesion. Because a minority of patients presented lesions attributable to more than one clinical form, the patient-level clinical form was defined by the presence of any multiple-inoculation lesion, which took precedence over other forms; the scar-level clinical form corresponds to the form recorded for the lesion that gave rise to each scar.

Scar assessment

Each scar was evaluated individually according to trophism (atrophic, normotrophic or hypertrophic) and specific morphology (linear, “wax drop” or nonspecific). Additional variables included anatomical site, clinical form of sporotrichosis, presence of inoculation chancre and orientation of the lesion axis relative to skin tension lines.

Trophism was assessed clinically by inspection and palpation, using the plane of the adjacent unaffected skin as the reference: scars depressed below this plane were classified as atrophic, those level with it as normotrophic, and those raised above it as hypertrophic. The “wax drop” pattern was defined according to the description by Lyra et al. [11], as a scar in which atrophic and hypertrophic areas are intermingled, conferring the distinctive appearance from which the pattern takes its name. Because this pattern is characterized by the coexistence of atrophic and hypertrophic components within a single scar, scars displaying it were classified as hypertrophic for the purposes of trophism analysis. Trophism and morphology were therefore not classified independently: all “wax drop” scars are included among the scars categorized as hypertrophic. Linear scars were defined as elongated lesions distributed along the lymphocutaneous axis. Each scar was initially assessed by a single dermatologist; in cases of doubt, it was additionally evaluated by two further dermatologists, and the final classification was established by consensus among them.

Cryotherapy

Cryotherapy was performed with liquid nitrogen using an open spray technique, with two freeze-thaw cycles of 30 seconds per session, applied monthly until lesion resolution or treatment interruption based on clinical judgment. It was used as an adjunctive measure at the discretion of the attending dermatologist, in accordance with the Brazilian Society of Dermatology recommendations for the management of human sporotrichosis [9], which support its use in verrucous and vegetating lesions and in lesions refractory to conventional antifungal therapy. In total, six patients received cryotherapy (two treated with cryotherapy alone and four with combined oral antifungal plus cryotherapy), from whose lesions the 38 scars analyzed in this subgroup arose. Scars arising from lesions treated with cryotherapy were analyzed separately.

Statistical analysis

Categorical variables were expressed as absolute values and percentages. Continuous variables were summarized using means, medians and standard deviations. Statistical analyses were performed using Microsoft Excel (Microsoft Corporation, Redmond, WA, USA), with confirmatory re-analysis in Python 3 (SciPy 1.x). Associations between categorical variables were assessed using Pearson’s chi-square test, and continuous variables were compared using the Mann-Whitney U test. A p-value < 0.05 was considered statistically significant.

Because most patients presented with more than one scar, and the number of scars per patient was highly uneven, observations at the scar level were not independent. Comparisons performed at the scar level are therefore reported as descriptive measures of association rather than as formal inferential tests. For variables defined at the patient level rather than at the scar level, namely treatment duration and, in the analysis of exposure to cryotherapy, the proportion of atrophic scars per patient, analyses were additionally performed with the patient as the unit of analysis, and these patient-level results are the ones considered informative.

Ethics statement

This study was approved by the Research Ethics Committee of Santa Casa de São Paulo School of Medical Sciences (CAAE: 88013323.1.0000.5479). Due to the retrospective nature of the study, the requirement for written informed consent was waived. Clinical images were used in accordance with institutional ethical standards and patient confidentiality was preserved.

## Results

Patient characteristics

Forty-five patients were included, with ages ranging from 2 to 83 years (mean 35.9 ± 23.8 years). Women accounted for 29 (64.4%) of the sample. The most frequent clinical forms were lymphocutaneous (22, 48.9%) and multiple inoculation (13, 28.9%). Detailed demographic and clinical characteristics are presented in Table 1.

**Table 1:** Demographic and clinical characteristics of patients with cutaneous sporotrichosis Demographic data, clinical forms of sporotrichosis, treatment modality, treatment duration and distribution of scars per patient among the 45 patients included in the study. Continuous variables are presented as mean ± standard deviation (range), and categorical variables as absolute numbers and percentages. All values are calculated with the patient as the unit of analysis.

Characteristic	n (%) or value
Number of patients	45
Female sex	29 (64.4%)
Age (years)	35.9 ± 23.8 (range: 2–83)
Clinical form	
Lymphocutaneous	22 (48.9%)
Multiple inoculation	13 (28.9%)
Localized cutaneous	9 (20.0%)
Systemic	1 (2.2%)
Treatment modality	
Oral antifungal therapy only	39 (86.7%)
Cryotherapy only	2 (4.4%)
Combined therapy (oral + cryotherapy)	4 (8.9%)
Treatment duration (weeks)	20.4 ± 14.7
Scars per patient, median (IQR)	2 (1–5); range 1–25

The 202 scars were unevenly distributed across the 45 patients (mean 4.5 ± 5.6 scars per patient; median 2, interquartile range 1-5, range 1-25). Fourteen patients (31.1%) contributed a single scar, whereas the five most affected patients (11.1% of the sample) accounted for 94 scars (46.5% of all scars analyzed). The full distribution of scars across patients is presented in Table 2.

**Table 2:** Distribution of scars per patient (n = 45 patients, 202 scars) Distribution of the 202 analyzed scars across the 45 patients included in the study. Median 2 scars per patient (interquartile range 1–5; range 1–25).

Scars per patient	Patients, n (%)
1	14 (31.1)
2	10 (22.2)
3	5 (11.1)
4	3 (6.7)
5	4 (8.9)
6	2 (4.4)
7	1 (2.2)
8	1 (2.2)
15	1 (2.2)
16	1 (2.2)
17	1 (2.2)
21	1 (2.2)
25	1 (2.2)
Total	45 (100)

Overall scar characteristics

A total of 202 scars were analyzed. Most were located on the upper limbs (162, 80.2%), followed by the head and neck (15, 7.4%), the lower limbs (10, 5.0%), the phalanges (8, 4.0%) and the trunk (7, 3.5%). Regarding trophism, 104 (51.5%) were atrophic, 91 (45.0%) hypertrophic, and 7 (3.5%) normotrophic. Linear scars accounted for 43 (21.3%) and the “wax drop” pattern for 37 (18.3%), the remaining 122 (60.4%) being nonspecific. The overall distribution of scar trophism, morphology and anatomical location is detailed in Table 3.

**Table 3:** Distribution of scars according to trophism, morphology and anatomical location (n = 202 scars) Overall distribution of scar trophism (atrophic, hypertrophic and normotrophic), morphological patterns (linear, “wax drop” and nonspecific), and anatomical location of scars evaluated after clinical cure of cutaneous sporotrichosis. All “wax drop” scars are also counted among the hypertrophic scars (see Methods).

Scar characteristic	n (%)
Scar trophism	
Atrophic	104 (51.5%)
Hypertrophic	91 (45.0%)
Normotrophic	7 (3.5%)
Scar morphology	
Linear	43 (21.3%)
“Wax drop” pattern	37 (18.3%)
Nonspecific	122 (60.4%)
Anatomical location	
Upper limbs (excluding hands)	162 (80.2%)
Phalanges	8 (4.0%)
Head and neck	15 (7.4%)
Lower limbs	10 (5.0%)
Trunk	7 (3.5%)

At the scar level, trophism was associated with the presence of an inoculation chancre (p = 0.00026) and with orientation of the lesion axis relative to skin tension lines (p < 0.001). Hypertrophic scars were more frequent in lesions arising from inoculation chancres (39 of 65, 60.0%, vs. 52 of 137, 37.9%) and in lesions whose axes were parallel to tension lines (56 of 76, 73.7%, vs. 35 of 126, 27.8%). Both associations persisted when the analysis was restricted to the contrast between atrophic and hypertrophic scars (p = 0.0011 and p < 0.001, respectively) and when hypertrophic scars were compared with all others (p = 0.0053 and p < 0.001).

A statistical association between clinical form and trophism was also detected (p = 0.0018). However, decomposition of the chi-square showed that 88.1% of its value derived from the normotrophic column, which comprised only seven scars distributed across cells with expected frequencies below 5.5, most notably four normotrophic scars in the multiple-inoculation group against an expected 1.0, and a single normotrophic scar in the lymphocutaneous group against an expected 5.5. The proportion of hypertrophic scars did not differ significantly across clinical forms (localized cutaneous 4 of 13, 30.8%; lymphocutaneous 72 of 158, 45.6%; multiple inoculation 14 of 28, 50.0%; systemic 1 of 3, 33.3%; p = 0.67). Clinical form was therefore not associated with hypertrophic scarring in this cohort, and the overall association should not be interpreted as such. These associations are summarized in Table 4 and, for the reasons detailed in the Statistical analysis section and the Limitations, are presented as descriptive.

**Table 4:** Association between clinical and topographical variables and scar trophism (n = 202 scars) Association between scar trophism and clinical form of sporotrichosis, presence of inoculation chancre, orientation of lesions relative to skin tension lines, and use of cryotherapy. Associations were assessed using Pearson’s chi-square test. All analyses were performed at the scar level; because scars were unevenly clustered within patients, the p-values are descriptive and do not constitute formal inferential tests. Several cells had expected frequencies below five, which further limits the reliability of the chi-square approximation. * For clinical form, 88.1% of the chi-square value derives from the normotrophic column (n = 7); the proportion of hypertrophic scars did not differ across clinical forms (p = 0.67), so this association should not be read as one with hypertrophic scarring (see Results). The cryotherapy comparison was not confirmed at the patient level (see Results). (-) indicates data not applicable.

Variable	Atrophic n (%)	Normotrophic n (%)	Hypertrophic n (%)	Total n (%)	p value
Clinical form					0.0018*
Lymphocutaneous	85 (53.8)	1 (0.6)	72 (45.6)	158 (78.2)	-
Localized cutaneous	7 (53.8)	2 (15.4)	4 (30.8)	13 (6.4)	-
Systemic	2 (66.7)	0 (0.0)	1 (33.3)	3 (1.5)	-
Multiple inoculation	10 (35.7)	4 (14.3)	14 (50.0)	28 (13.9)	-
Total	104 (51.5)	7 (3.5)	91 (45.0)	202 (100)	-
Presence of inoculation chancre					0.00026
Yes	21 (32.3)	5 (7.7)	39 (60.0)	65 (32.2)	-
No	83 (60.6)	2 (1.5)	52 (37.9)	137 (67.8)	-
Total	104 (51.5)	7 (3.5)	91 (45.0)	202 (100)	-
Orientation relative to skin tension lines					< 0.001
Parallel	20 (26.3)	0 (0.0)	56 (73.7)	76 (37.6)	-
Not parallel	84 (66.7)	7 (5.6)	35 (27.8)	126 (62.4)	-
Total	104 (51.5)	7 (3.5)	91 (45.0)	202 (100)	-
Cryotherapy					0.0076
With cryotherapy	28 (73.7)	0 (0.0)	10 (26.3)	38 (18.8)	-
Without cryotherapy	76 (46.3)	7 (4.3)	81 (49.4)	164 (81.2)	-
Total	104 (51.5)	7 (3.5)	91 (45.0)	202 (100)	-

Age and scar trophism

Scar trophism differed across patient age groups at the scar level (p = 0.036). Hypertrophic scars predominated among patients under 18 years (19 of 32, 59.4%) and in those aged 65 years or over (11 of 18, 61.1%) and accounted for half of the scars in the 40-64 age group (41 of 82, 50.0%), whereas atrophic scars predominated in the 18-39 age group (47 of 70, 67.1%). Normotrophic scars were infrequent in all strata (one, three and three scars in the under-18, 18-39 and 40-64 groups, respectively, and none among patients aged 65 years or over). The 40-64 age group was also the most heavily clustered, with three patients contributing 62 of its 82 scars (75.6%). When the analysis was repeated at the patient level, the correlation between age and the proportion of hypertrophic scars per patient was not significant (Spearman's rho = -0.17; p = 0.26).

Scars arising from lesions treated with cryotherapy

Thirty-eight (18.8%) scars, arising from lesions in six patients, were treated with cryotherapy. In this group, 28 (73.7%) scars were atrophic and 10 (26.3%) hypertrophic, with no normotrophic scars identified. At the scar level, the proportion of atrophic scars was higher than in the non-cryotherapy group (p = 0.0076). However, this difference was concentrated in a small number of patients: two of the six patients exposed to cryotherapy contributed 23 of the 28 atrophic scars (82.1%) in this subgroup. When the patient was used as the unit of analysis, the mean proportion of atrophic scars per patient did not differ significantly between those exposed and not exposed to cryotherapy (59.4% vs. 45.2%; p = 0.45).

Treatment duration was a patient-level rather than a scar-level variable, as all scars from a given patient shared the same value. Analyzed per patient, the mean treatment duration was 30.8 ± 23.4 weeks among the six patients exposed to cryotherapy and 18.8 ± 12.6 weeks among the 39 patients not exposed, a difference that was not statistically significant (p = 0.27). Cryotherapy was therefore not associated with a shorter treatment duration in this cohort.

## Discussion

**Figure 1:**
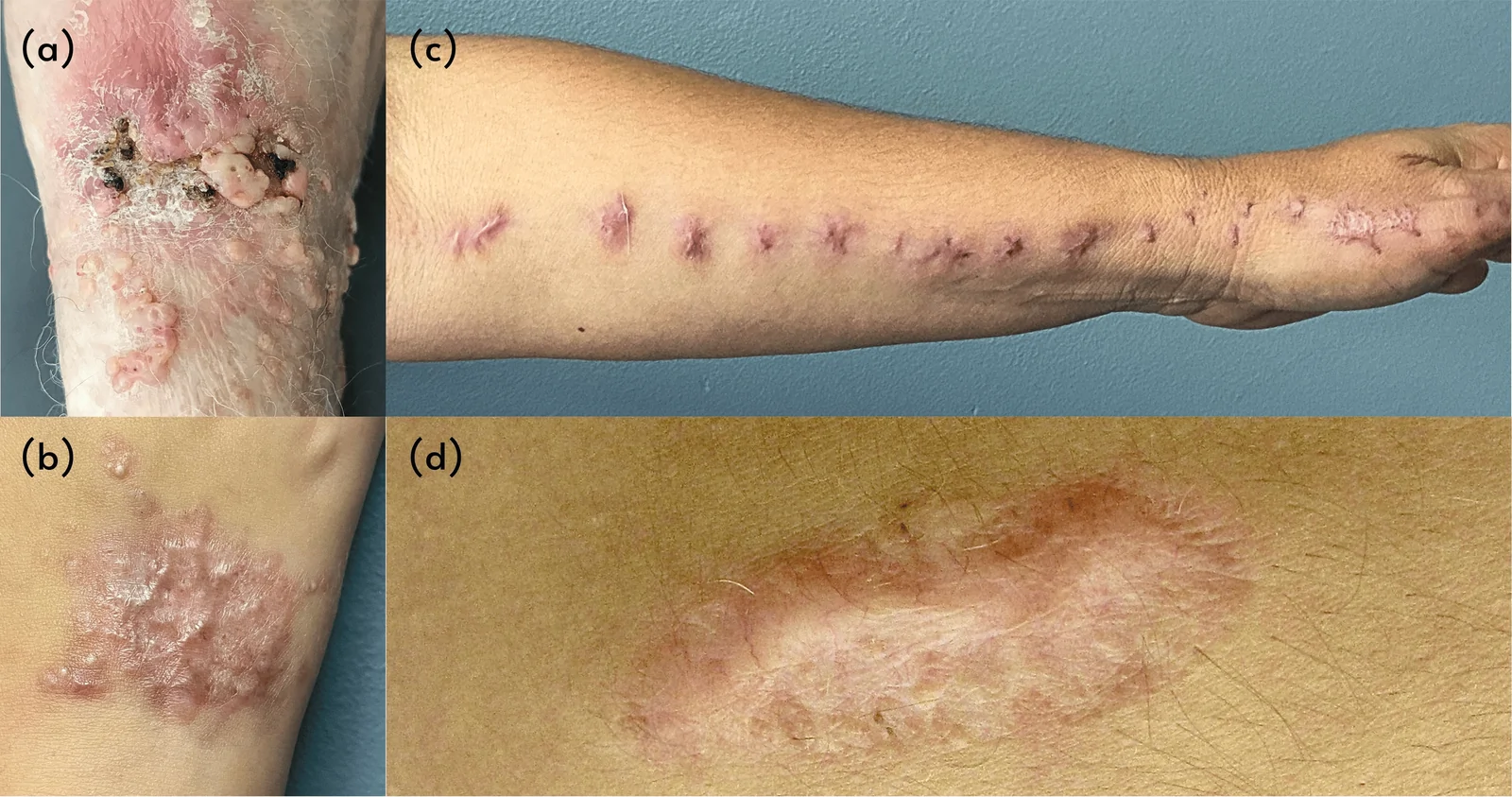
Representative “wax drop” and linear scarring patterns following cutaneous sporotrichosis Clinical photographs of patients treated for sporotrichosis at the Tropical Dermatoses Outpatient Clinic of the Dermatology Department, Santa Casa de São Paulo, illustrating “wax drop” scars (a and b) and linear scars (c and d).

**Figure 2:**
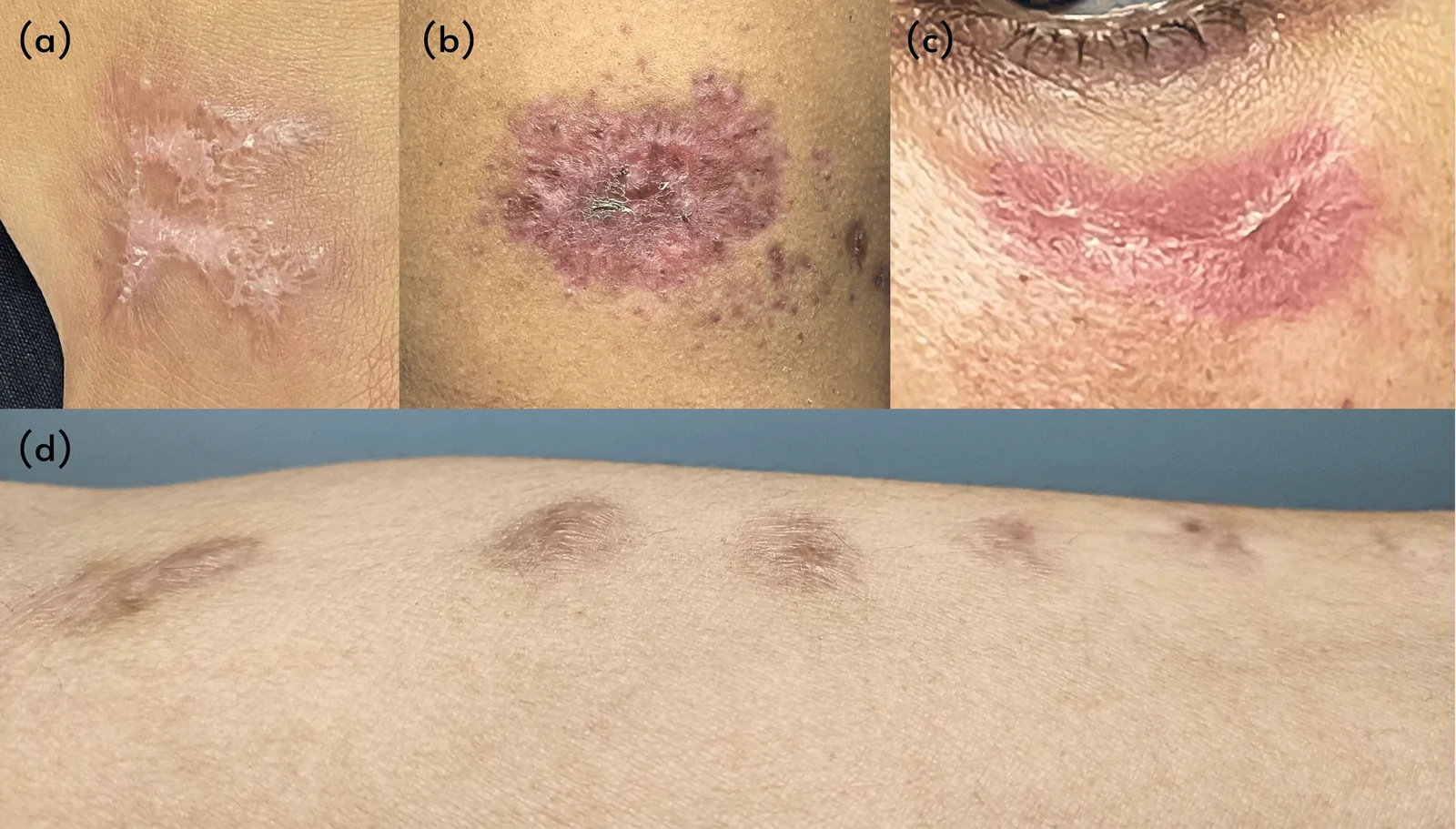
Representative scar morphologies following cutaneous sporotrichosis Clinical photographs of patients treated for sporotrichosis at the Tropical Dermatoses Outpatient Clinic of the Dermatology Department, Santa Casa de São Paulo, illustrating different scar patterns observed after clinical cure (a-d).

Representative “wax drop” and linear scarring patterns are illustrated in Figure 1. This study shows that cutaneous sporotrichosis may result in a substantial burden of scarring, often with distinctive morphological patterns, as shown in Figure 2. More than half of the scars were atrophic, but hypertrophic scars were also frequent, highlighting the heterogeneous nature of wound healing in this infection [11].

An aspect with important clinical and psychosocial implications is the frequent linear arrangement of scars, particularly on the upper limbs. This distribution and morphology may mimic scars resulting from non-suicidal self-injury, potentially leading to misinterpretation by healthcare professionals, educators or family members, and exposing patients to stigma or inappropriate assumptions. The psychosocial impact of visible scars is well documented, and misattribution may further exacerbate quality-of-life impairment [5,12]. In our view, this observation, which does not depend on inferential testing, represents the most directly actionable finding of the present series.

The association between hypertrophic scarring and the presence of an inoculation chancre, the site of initial fungal implantation, where local inflammation is earliest and most intense, is compatible with the concept that inflammatory burden contributes to fibrotic repair [13]. We had initially expected this reasoning to extend to the more disseminated clinical forms, and a statistical association between clinical form and trophism was indeed present. On closer analysis, however, that association was generated almost entirely by the uneven distribution of seven normotrophic scars across sparse cells, and the proportion of hypertrophic scars did not differ across clinical forms. Clinical form therefore does not support an inflammatory interpretation in this cohort, and we do not advance one. Any such interpretation here rests on the inoculation chancre alone and remains speculative, as the analyses were descriptive and unadjusted.

Lesion orientation along skin tension lines was associated with hypertrophic scarring at the scar level, a pattern that contrasts with classic surgical wound healing paradigms. In elective surgical practice, incisions made parallel to Langer’s lines are associated with lower rates of hypertrophic scarring [14,15]. One possible interpretation is that, in the context of infectious dermatoses such as sporotrichosis, inflammatory burden and pathogen-related factors may override biomechanical advantages typically conferred by lesion orientation. This hypothesis merits testing in a prospective design, and the present data are not sufficient to challenge the established surgical concept.

Cryotherapy has been reported as an effective adjunctive treatment in sporotrichosis [16], and improvements in hypertrophic scar quality with energy-based devices support the broader concept that therapeutic interventions can influence dermal remodeling beyond infection control [17]. In our cohort, scars arising from lesions treated with cryotherapy were more frequently atrophic at the scar level. However, this difference was not confirmed when the patient was used as the unit of analysis, and it was concentrated in two of the six exposed patients, who together contributed 82% of the atrophic scars in that subgroup. Similarly, the apparent difference in treatment duration observed at the scar level did not persist at the patient level, where treatment duration is properly defined. We therefore do not interpret these observations as evidence that cryotherapy influences scar trophism or shortens treatment. With only six exposed patients, this cohort is underpowered to address the question, and the indication of cryotherapy for verrucous, vegetating or refractory lesions [9] introduces confounding by indication that cannot be resolved retrospectively. It should also be noted that cryotherapy may itself induce pigmentary alterations - particularly post-inflammatory hypo- or hyperpigmentation in patients with darker phototypes - which would need to be weighed in any assessment of its cosmetic benefit.

Limitations

The retrospective design and single-center setting limit the generalizability of the findings. The decision to perform cryotherapy was based on clinical judgment and lesion characteristics, which introduces treatment selection bias and confounding by indication. The study does not allow causal inference regarding the effect of cryotherapy on scar outcomes, and with six exposed patients it is underpowered for that question.

The principal limitation concerns the unit of analysis. The number of scars per patient ranged from 1 to 25 (median 2, interquartile range 1-5), and the five most affected patients contributed 94 of the 202 scars (46.5%). Because the analyses summarized in Table 4 were performed at the scar level, observations were not independent, and a small subset of patients, together with their individual biological determinants of wound healing, exerted a disproportionate influence on the estimates. Using Kish’s approximation, the weighted mean cluster size is 11.2; for an intraclass correlation of 0.10-0.20, a plausible range for wound-healing phenotypes, the effective sample size would be approximately 66-100 scars rather than 202. The reported p-values are therefore descriptive rather than formal inferential tests, and the associations are hypothesis-generating. Analyses accounting for within-patient clustering, such as generalized estimating equations or mixed-effects models, would be required to confirm them. In addition, several cells in Table 4 had expected frequencies below five, which limits the chi-square approximation; exact methods would be preferable in a larger series.

Patient age, a recognized modulator of wound healing, was associated with scar trophism at the scar level (p = 0.036) but not at the patient level, and was not adjusted for; residual confounding by age therefore cannot be excluded. Skin phototype and pigmentary sequelae (post-inflammatory hyper- or hypopigmentation, including cryotherapy-induced dyschromia) were not recorded; this is particularly relevant in the ethnically diverse Brazilian population studied, where phototype influences both healing and cosmetic outcome. In addition, the timing of scar assessment after clinical cure was not standardized, and scar morphology may evolve over time. Finally, analyses were univariate, without multivariable modelling, so it cannot be determined whether the reported associations are independent of one another.

Scar assessment relied on clinical and photographic evaluation, which may involve some degree of subjectivity, and histological or molecular analyses of scars were not performed. No validated scar assessment instrument, such as the Vancouver Scar Scale or the Patient and Observer Scar Assessment Scale, was applied; classification was based on inspection and palpation relative to the plane of adjacent skin. Moreover, because duplicate assessment was undertaken only for scars considered doubtful by the initial evaluator, inter-observer agreement could not be formally quantified. Finally, the “wax drop” pattern, which by definition combines atrophic and hypertrophic areas within the same scar [11], was assigned to the hypertrophic category by convention. These 37 scars account for 40.7% of the 91 classified as hypertrophic, so the reported trophism distribution is contingent on this rule; had they been treated as a separate mixed category, hypertrophic scars would have represented 54 (26.7%) rather than 91 (45.0%) of the sample. Trophism and morphology were consequently not independent classifications, and the associations involving hypertrophic trophism partly reflect the distribution of the “wax drop” morphology. Future studies may benefit from applying a validated scar scale and from recording mixed-trophism scars as a distinct category.

## Conclusions

Cutaneous sporotrichosis is associated with a substantial scar burden, frequently presenting with distinctive morphological patterns such as linear and "wax drop" scars. The frequent linear arrangement of these scars on the upper limbs, which may be mistaken for self-inflicted injury, is a clinically actionable observation that warrants awareness among clinicians caring for these patients. At the scar level, the presence of an inoculation chancre and a lesion axis parallel to skin tension lines were both associated with hypertrophic scarring and were robust to sensitivity analyses, whereas a statistical association with clinical form was driven by a small number of normotrophic scars and did not extend to hypertrophic scarring. Given the marked and uneven clustering of scars within patients, all of these associations should be regarded as descriptive and hypothesis-generating rather than inferential.

Differences in scar trophism and treatment duration associated with cryotherapy, observed at the scar level, were not confirmed when the patient was used as the unit of analysis and were driven by a small number of patients; they should not be interpreted as evidence of a therapeutic effect. Taken together, these findings indicate that scarring deserves greater attention as a clinical outcome in sporotrichosis, both for patient counseling and for the design of future prospective studies with clustering-adjusted analyses and controlled treatment allocation.
